# Analytical agreement and clinical interchangeability of routine complete blood count parameters between the Atellica HEMA 580 and Sysmex XN-1000 analyzers: a CLSI EP09c study

**DOI:** 10.3389/fmed.2026.1878534

**Published:** 2026-07-02

**Authors:** Quang Tung Nguyen, Van Tuan Pham, Chi Thanh Nguyen, Phuc Minh Hoang Nguyen, Dieu Huong Nguyen, Huong Lan Tong

**Affiliations:** 1 Department of Hematology, Hanoi Medical University, Hanoi, Vietnam; 2 Department of Hematology and Transfusion Medicine, Hanoi Medical University Hospital, Hanoi, Vietnam; 3 Department of Hematology Laboratory, Hanoi Medical University, Hanoi, Vietnam

**Keywords:** Atellica HEMA 580, automated hematology analyzer, CLSI EP09c, complete blood count, method comparison, Sysmex XN-1000

## Abstract

**Background:**

Analytical consistency in complete blood count (CBC) testing is essential when introducing new hematology analyzers into routine clinical use. This study evaluated the agreement between the Atellica HEMA 580 and the Sysmex XN-1000 using a standardized method-comparison approach.

**Methods:**

A total of 100 residual K₂EDTA whole-blood samples were analyzed on both systems between April and June 2025. Method comparison followed CLSI EP09c guidelines, including Passing–Bablok regression, Spearman correlation, bias estimation at predefined medical-decision levels, and Bland–Altman analysis.

**Results:**

Strong analytical agreement was observed for most parameters, with correlation coefficients exceeding 0.98 for key indices including RBC (*r* = 0.999), HGB (*r* = 0.998), WBC (*r* = 0.996), and PLT-I (*r* = 0.995), and regression slopes close to unity. Bias estimates at medical-decision levels were within allowable limits for the majority of parameters. WBC at 0.5 × 10^9^/L and PLT-I at 100 × 10^9^/L achieved Outcome A, indicating optimal agreement at clinically critical thresholds. In contrast, MCV showed proportional deviation with bias exceeding allowable limits at both 80 fL and 100 fL, and RDW-CV demonstrated lower correlation (*r* = 0.848) and greater dispersion. Bland–Altman analysis confirmed small mean bias for most parameters, with narrow limits of agreement for RBC (−1.48%, LoA − 4.19 to 1.36) and HGB (1.54%, LoA − 1.37 to 4.19), while wider limits were observed for WBC (−12.62 to 1.70) and PLT-I (−16.55 to 9.51). RDW-CV exhibited the broadest limits of agreement (−17.76 to 10.38). Among differential parameters, lymphocyte count showed optimal agreement (bias −1.12%, Outcome A), while other indices remained within acceptable ranges.

**Conclusion:**

The Atellica HEMA 580 shows acceptable analytical agreement with the Sysmex XN-1000 for RBC, HGB, WBC, PLT-I, and the evaluated neutrophil and lymphocyte differential parameters, and can be applied in clinical laboratory practice. In contrast, MCV and RDW-CV demonstrated analyzer-dependent differences that warrant cautious interpretation when comparing results across platforms or during longitudinal assessment.

## Introduction

Complete blood count (CBC) testing is one of the most commonly requested laboratory investigations and plays a central role in the diagnosis, classification, and monitoring of a wide range of hematological and systemic conditions (1, 2). Quantitative assessment of white blood cells (WBC), red blood cells (RBC), and platelets (PLT) provides critical information for clinical decision-making in settings such as infection, anemia, bleeding disorders, and malignancy. With increasing laboratory workload and demand for rapid turnaround times, modern hematology analyzers have evolved to deliver high-throughput, automated measurements with improved analytical precision and standardization. These systems typically integrate electrical impedance with optical or fluorescence-based flow cytometry, enabling detailed cellular characterization and reducing the need for manual microscopic review (3).

The Sysmex XN-1000 (Sysmex Corporation, Kobe, Japan) is a well-established hematology analyzer that employs impedance and fluorescence flow cytometry for cell counting and differential analysis, and is widely used in routine clinical laboratories (2). The Atellica HEMA 580 (Siemens Healthcare Diagnostics Inc., Tarrytown, NY, USA) is a newer analytical platform designed for medium- to high-throughput environments, incorporating comparable measurement principles for CBC, extended leukocyte differentiation, reticulocyte analysis, and automated slide preparation. Although initial analytical evaluations of the Atellica HEMA 580 have been reported (4), evidence regarding its comparability with established systems in real-world laboratory settings remains limited. Given that different analyzers may vary in calibration, signal processing, and algorithm-based data interpretation, even small systematic differences can affect the classification of results near clinical decision thresholds.

Because CBC parameters are frequently used for diagnosis, treatment monitoring, and longitudinal patient follow-up, ensuring consistency between analytical systems is essential when introducing a new platform into routine practice. Discrepancies between instruments may lead to misinterpretation of trends or inappropriate clinical decisions, particularly in critical values or borderline results. While existing guidelines such as CLSI H26-A2 (5), ICSH recommendations (1), and other evaluation frameworks (6) provide guidance on analytical validation and quality assurance, they do not fully address method comparability using patient samples. In this context, the CLSI EP09c guideline offers a structured approach for method-comparison studies by combining regression analysis, Bland–Altman assessment, and bias estimation at clinically relevant decision levels (7), allowing interpretation of analytical differences in terms of clinical acceptability. Therefore, this study was conducted to evaluate the analytical agreement of routine CBC parameters and selected clinically relevant leukocyte differential indices between the Atellica HEMA 580 and the Sysmex XN-1000 using the CLSI EP09c method-comparison framework.

## Materials and methods

### Study design and setting

This method-comparison study was conducted at the Department of Hematology and Transfusion Medicine, Hanoi Medical University Hospital. The study was performed using residual anonymized blood samples collected for routine complete blood count (CBC) testing between April and June 2025.

### Samples

Residual venous blood samples submitted for routine CBC testing during the study period were included in the analysis. A total of 100 K₂EDTA-anticoagulated whole-blood specimens were included. Blood samples were collected into standard EDTA tubes (Changsha Renji Medical Equipment Co., Ltd., Changsha, China) and were excluded if they showed visible clots, hemolysis, or insufficient volume. All specimens were processed under uniform laboratory conditions and analyzed on both analyzers within 4 h of venipuncture, with samples stored at room temperature (20–25 °C) prior to measurement. Before analysis, each sample was gently mixed by inversion according to manufacturer recommendations to ensure homogeneity. To minimize pre-analytical variability, all measurements were performed by a single trained technologist following standardized procedures. Patient identifiers were removed prior to analysis, and only anonymized data were used for statistical evaluation.

In accordance with the CLSI EP09c guideline for method-comparison studies, approximately 100 patient specimens distributed across the analytical measurement range were included. The primary objective of the study was to evaluate analytical agreement between measurement procedures rather than to estimate population parameters; therefore, a formal statistical power calculation was not performed. To ensure adequate representation across clinically relevant concentration ranges, samples with low, normal, and high values encountered during routine laboratory testing were retained whenever available. The resulting dataset covered broad measurement intervals for key parameters, including RBC (2.56–7.10 × 10^12^/L), HGB (2.7–15.7 g/dL), WBC (0.64–28.72 × 10^9^/L), and PLT-I (6.5–1010.5 × 10^9^/L), thereby encompassing clinically relevant low, normal, and high concentrations. In addition, specimens representing important decision ranges such as severe anemia, leukopenia, and thrombocytopenia were intentionally included whenever encountered to facilitate evaluation near clinically relevant thresholds.

### Analyzers

Two automated hematology analyzers were evaluated: the Sysmex XN-1000 (Sysmex, Kobe, Japan) and the Atellica HEMA 580 (Siemens Healthcare Diagnostics Inc., Tarrytown, NY, USA). Both instruments were operated using manufacturer-recommended reagents, diluents, and calibrators in accordance with standard operating procedures. Internal quality control was performed daily prior to sample analysis using tri-level commercial control materials (XN-Check, Sysmex; Atellica Hematology 3-in-1 Controls, Siemens), and testing was initiated only when control results met predefined acceptance criteria. Instrument calibration was conducted at installation and subsequently verified at regular intervals, including after reagent lot changes, in line with the manufacturers’ recommendations.

### Analytical principles

Both analyzers perform complete blood count (CBC) measurements and six-part white blood cell (WBC) differentials using a combination of impedance and fluorescence flow cytometry, although their optical configurations and data-processing algorithms differ.

The Sysmex XN-1000 uses impedance for red blood cell (RBC) and platelet (PLT) counting, and spectrophotometry for hemoglobin (HGB) measurement. Leukocyte differentiation and reticulocyte analysis are based on fluorescence flow cytometry with hydrodynamic focusing, allowing separation of cell populations according to forward scatter, side scatter, and fluorescence intensity. In addition to the impedance-based platelet count (PLT-I), the system includes an optical platelet channel (PLT-F) that improves accuracy at low platelet concentrations (2, 8, 9). In this study, only PLT-I results were used for comparison.

The Atellica HEMA 580 similarly measures RBCs and PLTs by impedance and determines HGB concentration by spectrophotometry at 555 nm in the total nucleated cell (TNC)/HGB chamber. WBC analysis is performed across three measurement channels, including the TNC/HGB, BASO/TNC2, and LMNE chambers. The final WBC count is derived from combined signals from the TNC/HGB and LMNE channels, while the BASO/TNC2 channel functions as a reference for basophil enumeration and detection of nucleated red blood cells (NRBCs). Differential leukocyte analysis is carried out using a dual-channel system integrating impedance and optical detection with double hydrodynamic sequential flow (DHSS). Basophils are measured in the BASO channel, whereas other WBC subpopulations and immature cells are characterized in the LMNE chamber (4, 10).

### Data analysis

Comparability between the Atellica HEMA 580 and Sysmex XN-1000 analyzers was assessed in accordance with the CLSI EP09c guideline (7). Statistical analyses included Passing–Bablok regression, Spearman’s rank correlation analysis, bias estimation at predefined medical decision levels, and Bland–Altman difference plots. Passing–Bablok regression was used to evaluate systematic differences between methods, with 95% confidence intervals (CIs) of the intercept and slope applied to detect constant and proportional bias, respectively. Constant bias was considered present when the 95% CI of the intercept did not include 0, whereas proportional bias was considered present when the 95% CI of the slope did not include 1.0. Spearman’s rank correlation coefficient (*ρ*) was used *a priori* for all evaluated parameters to provide a uniform nonparametric measure of association across the entire method-comparison dataset. This approach was selected because several parameters demonstrated non-normal distributions, unequal distribution across measurement ranges, and potential susceptibility to outliers. Using a single correlation method for all parameters ensured analytical consistency and avoided parameter-specific selection of correlation tests. Correlation coefficients ≥0.90 were interpreted as indicating very strong correlation (11). Bias at clinically relevant decision levels was estimated using regression equations with 95% confidence intervals (CIs) obtained by nonparametric bootstrap resampling (1,000 iterations). For each bootstrap sample, the regression-derived bias at the predefined medical-decision level was recalculated, and the 2.5th and 97.5th percentiles of the resulting empirical distribution were used to derive the 95% CI. Thus, confidence intervals were percentile-based rather than bias-corrected and accelerated (BCa) intervals, consistent with the default MedCalc implementation. Because some parameters, particularly HGB, demonstrated extremely small variability around the regression estimate, the resulting bootstrap confidence intervals were very narrow and could appear identical after rounding (12). The predefined medical-decision levels were selected to represent clinically important conditions, including severe anemia (HGB 7 g/dL), leukopenia (WBC 0.5 × 10^9^/L), neutropenia (neutrophil count 0.5 × 10^9^/L), and thrombocytopenia (PLT-I 50 and 100 × 10^9^/L). Analytical performance was classified into five categories (A–E) according to CLSI EP09c criteria, where A indicates optimal agreement, B–C acceptable agreement, and D–E unacceptable bias. Bland–Altman analysis was performed to evaluate agreement by estimating mean bias and 95% limits of agreement (LoA), defined as mean ± 1.96 standard deviations of the differences. The Shapiro–Wilk test was used to assess normality of bias distribution; mean or median bias values were reported accordingly. Importantly, the Shapiro–Wilk analysis was applied to the distributions of percentage differences used in Bland–Altman analysis and was not used as a criterion for selecting the correlation method. Because statistically significant bias may not necessarily indicate clinical non-interchangeability, regression and Bland–Altman results were interpreted in conjunction with predefined allowable bias specifications derived from biological variation. Passing–Bablok regression was performed using the standard nonparametric implementation available in MedCalc Statistical Software, with estimation of slope and intercept and their corresponding 95% confidence intervals according to the default MedCalc algorithm. All statistical analyses were conducted using MedCalc Statistical Software (version 22.017; MedCalc Software Ltd., Ostend, Belgium).

The analytical comparison focused on selected CBC parameters, including WBC, RBC, HGB, PLT-I, MCV, RDW-CV, and representative leukocyte differential parameters (neutrophil and lymphocyte counts and percentages), based on their established clinical relevance, frequent use in routine hematological assessment, and adequate representation across the analytical measurement range of the study specimens.

Although both analyzers provide a complete five-part leukocyte differential, monocyte, eosinophil, and basophil parameters were not included in the predefined formal comparison. These leukocyte subpopulations generally occur at lower absolute concentrations in routine patient samples and were insufficiently represented across clinically relevant concentration ranges in the present dataset. Consequently, reliable application of CLSI EP09c regression-based agreement analyses and bias estimation at medical-decision levels could not be ensured for these parameters. To avoid potentially unstable or misleading analytical conclusions arising from sparse data distribution, the predefined comparison was restricted to neutrophil and lymphocyte parameters, which were adequately represented throughout the measurement range.

## Results

### Passing-Bablok regression and correlation analysis

Evaluated parameters included WBC, RBC, HGB, PLT-I, mean corpuscular volume (MCV), red cell distribution width (RDW-CV), and neutrophil and lymphocyte counts and percentages. Monocyte, eosinophil, and basophil parameters were not included in the predefined analytical comparison because the available specimen set contained limited representation across clinically relevant concentration ranges for these leukocyte subpopulations. Therefore, only neutrophil and lymphocyte differential parameters were included in the subsequent agreement analyses. Passing–Bablok regression analysis (Table 1) demonstrated a strong association between the two analyzers across all evaluated parameters, with Spearman’s rank correlation coefficients (*ρ*) ranging from 0.848 to 0.999. The highest correlations were observed for RBC (*ρ* = 0.999), HGB (*ρ* = 0.998), WBC (*ρ* = 0.996), and PLT-I (*ρ* = 0.995), whereas RDW-CV showed the lowest correlation (*ρ* = 0.848). No evidence of either constant or proportional bias was detected for RDW-CV, lymphocyte count, neutrophil percentage, or lymphocyte percentage based on the predefined regression criteria. In contrast, MCV demonstrated both constant and proportional bias, with a slope of 1.178 (95% CI: 1.102–1.248) and an intercept of −16.039 (95% CI: −22.06 to −9.57). WBC, neutrophil count, and PLT-I showed proportional bias without evidence of constant bias, whereas RBC and HGB demonstrated statistically detectable constant bias together with proportional bias for RBC. Despite these findings, the magnitude of deviation for most parameters remained small and was generally within clinically acceptable analytical limits. RDW-CV demonstrated comparatively broader confidence intervals and lower correlation than the other evaluated parameters, indicating greater analytical variability across the measurement range.

**Table 1:** Passing–Bablok regression analysis and agreement assessment of routine CBC parameters between the Atellica HEMA 580 and Sysmex XN-1000 analyzers.

Parameter	Atellica HEMA 580 Median (Range)	Sysmex XN-1000 Median (Range)	Passing–Bablok regression equation^†^	Intercept (95% CI)	Slope (95% CI)	Constant bias^‡^	Proportional bias^§^	Spearman’s *ρ* (95% CI)
RBC (×10^12^/L)	4.51 (2.63–7.00)	4.60 (2.56–7.10)	y = 0.154 + 0.949x	0.154 (0.116 to 0.191)	0.949 (0.941 to 0.959)	Present	Present	0.999 (0.998–0.999)
HGB (g/dL)	8.30 (2.7–15.7)	8.15 (2.7–15.7)	y = 0.100 + 1.000x	0.100 (0.098 to 0.102)	1.000 (0.998 to 1.002)	Present	Absent	0.998 (0.998–0.999)
MCV (fL)	90.2 (55.3–113.4)	90.3 (63.3–107.1)	y = −16.039 + 1.178x	−16.039 (−22.06 to −9.57)	1.178 (1.102 to 1.248)	Present	Present	0.951 (0.927–0.966)
RDW-CV (%)	13.45 (10.3–23.3)	14.00 (11.4–25.2)	y = 0.011 + 0.970x	0.011 (−1.222 to 1.378)	0.970 (0.869 to 1.058)	Absent	Absent	0.848 (0.782–0.895)
WBC (×10^9^/L)	7.34 (0.62–27.64)	7.54 (0.64–28.72)	y = 0.040 + 0.942x	0.040 (−0.081 to 0.161)	0.942 (0.928 to 0.962)	Absent	Present	0.996 (0.994–0.997)
Neutrophil count (×10^9^/L)	4.19 (0.32–21.84)	4.57 (0.31–24.02)	y = 0.039 + 0.908x	0.039 (−0.013 to 1.117)	0.908 (0.891 to 0.925)	Absent	Present	0.993 (0.990–0.995)
Lymphocyte count (×10^9^/L)	2.11 (0.10–13.51)	2.09 (0.08–14.37)	y = 0.034 + 0.968x	0.034 (−0.027 to 0.098)	0.968 (0.936 to 1.000)	Absent	Absent	0.977 (0.966–0.985)
Neutrophils (%)	59.5 (18.0–84.5)	61.2 (16.9–85.1)	y = −0.750 + 1.000x	−0.750 (−2.241 to 0.657)	1.000 (0.973 to 1.023)	Absent	Absent	0.983 (0.975–0.989)
Lymphocytes (%)	30.3 (6.1–57.6)	28.1 (6.3–58.2)	y = 1.300 + 1.000x	1.300 (0.585 to 2.256)	1.000 (0.969 to 1.025)	Present	Absent	0.982 (0.973–0.988)
PLT-I (×10^9^/L)	261 (5.5–1,006)	285 (6.5–1010.5)	y = 3.527 + 0.946x	3.527 (−1.076 to 7.165)	0.946 (0.928 to 0.973)	Absent	Present	0.995 (0.992–0.996)

CBC, complete blood count; RBC, red blood cell count; HGB, hemoglobin; MCV, mean corpuscular volume; RDW-CV, red cell distribution width coefficient of variation; WBC, white blood cell count; PLT-I, impedance platelet count. ^†^ Regression equations are expressed as Atellica HEMA 580 (y) versus Sysmex XN-1000 (x). ^‡^ Constant bias was considered present when the 95% confidence interval (CI) of the intercept did not include 0. ^§^Proportional bias was considered present when the 95% CI of the slope did not include 1.0.

**Figure 1:**
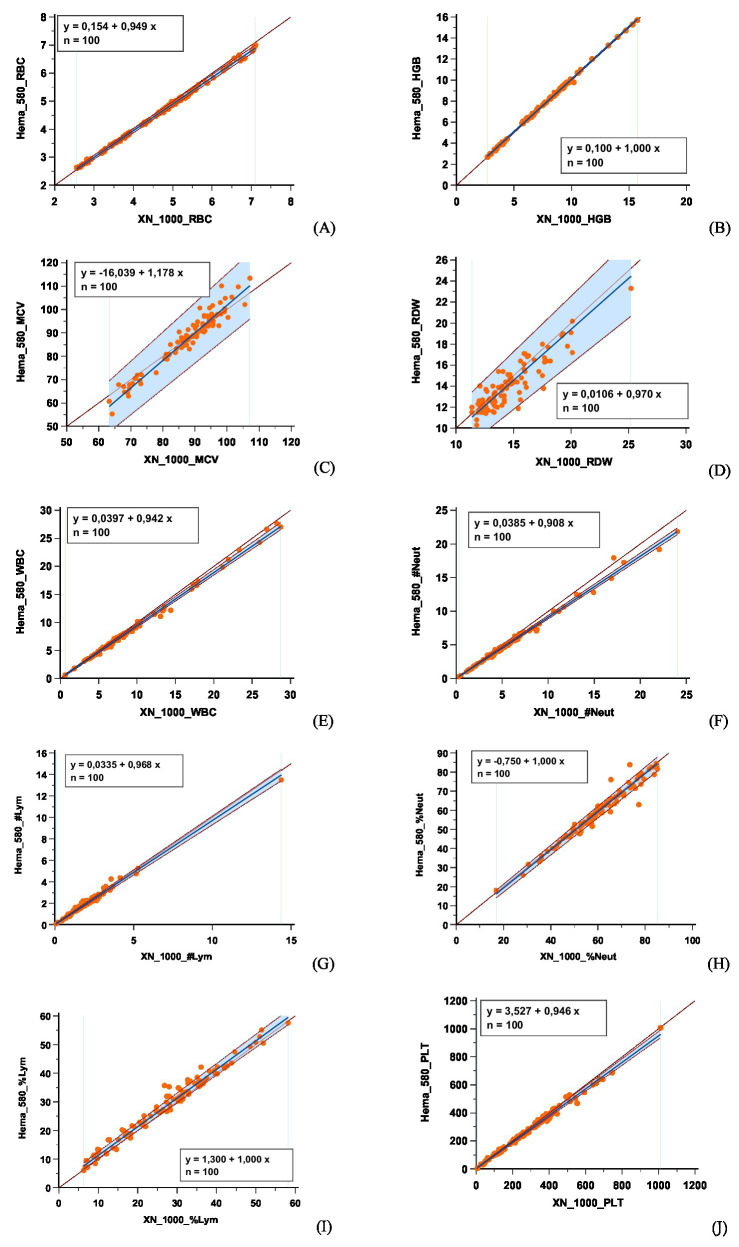
Passing–Bablok regression analysis comparing routine complete blood count (CBC) parameters measured on the Atellica HEMA 580 and Sysmex XN-1000 analyzers. Panels show comparisons for **(A)** RBC, **(B)** HGB, **(C)** MCV, **(D)** RDW-CV, **(E)** WBC, **(F)** neutrophil count, **(G)** lymphocyte count, **(H)** neutrophils (%), **(I)** lymphocytes (%), and **(J)** PLT-I. The solid line represents the Passing–Bablok regression line, and the dashed line represents the line of identity (y = x).

Figure 1 shows data points for all evaluated parameters were distributed in close proximity to the line of identity (y = x) across the full measurement range, including RBC (2.56–7.1 × 10^12^/L), HGB (2.7–15.7 g/dL), WBC (0.64–28.72 × 10^9^/L), PLT-I (6.5–1010.5 × 10^9^/L), neutrophil count (0.31–24.02 × 10^9^/L), lymphocyte count (0.08–14.37 × 10^9^/L), neutrophil percentage (16.9–85.1%), and lymphocyte percentage (6.3–58.2%). The scatterplots demonstrated a narrow dispersion around the regression lines for most parameters, consistent with the high correlation coefficients observed in Table 1 (*r* ≥ 0.982 for all except RDW-CV). In contrast, MCV (63.3–107.1 fL) and RDW-CV (11.4–25.2%) exhibited a wider spread of data points with greater deviation from the line of identity, corresponding to their comparatively lower correlation coefficients (MCV: *r* = 0.951; RDW-CV: *r* = 0.848) and broader variability across the measurement range.

### Bias estimation at medical-decision levels

When mean bias values were evaluated according to the desirable biological variation specifications from the EFLM database (Table 2), bias estimates were reported across predefined medical decision levels for each parameter. For HGB, bias values ranged from 0.5 to 1.43% at decision levels of 7, 10.5, 17, and 20 g/dL, all within the allowable limit of 2.5% and classified as Outcome B. For MCV, bias was −2.23% (95% CI: −3.4 to −1.4) at 80 fL and 1.78% (0.59 to 3.14) at 100 fL, both exceeding the allowable bias of 1.5% and classified as Outcome D. WBC showed bias values of 0.56% (−2.37 to 3.84) at 0.5 × 10^9^/L (Outcome A), −4.43% (−6.83 to −1.64) at 3 × 10^9^/L, −5.42% (−6.25 to −4.25) at 12 × 10^9^/L, and −5.62% (−6.75 to −3.97) at 30 × 10^9^/L, all within the allowable limit of 7.6% and classified as Outcomes A or B. Neutrophil count at 0.5 × 10^9^/L showed a bias of −1.54% (−10.29 to 12.61) against an allowable limit of 10.8%, corresponding to Outcome C. For PLT-I, bias values were 1.65% (−5.5 to 7.4) at 50 × 10^9^/L, −1.88% (−4.7 to 0.37) at 100 × 10^9^/L, −4.82% (−6.12 to −2.96) at 600 × 10^9^/L, and −5.05% (−6.55 to −2.89) at 1000 × 10^9^/L, with an allowable bias of 5.6%, corresponding to Outcomes C, A, C, and C, respectively.

**Table 2:** Bias estimates at predefined medical-decision levels and analytical performance classification according to CLSI EP09c criteria.

Parameter/medical-decision level	Estimated bias, % (95% CI)	Desirable allowable bias^*^	CLSI EP09c outcome^†^
HGB (g/dL), 7	1.43 (1.43 to 1.43)	2.5	B
HGB (g/dL), 10.5	0.95 (0.95 to 0.95)	2.5	B
HGB (g/dL), 17	0.59 (0.59 to 0.59)	2.5	B
HGB (g/dL), 20	0.50 (0.50 to 0.50)	2.5	B
MCV (fL), 80	−2.23 (−3.40 to −1.40)	1.5	D
MCV (fL), 100	1.78 (0.59 to 3.14)	1.5	D
WBC (×10^9^/L), 0.5	0.56 (−2.37 to 3.84)	7.6	A
WBC (×10^9^/L), 3	−4.43 (−6.83 to −1.64)	7.6	B
WBC (×10^9^/L), 12	−5.42 (−6.25 to −4.25)	7.6	B
WBC (×10^9^/L), 30	−5.62 (−6.75 to −3.97)	7.6	B
Neutrophil count (×10^9^/L), 0.5	−1.54 (−10.29 to 12.61)	10.8	C
PLT-I (×10^9^/L), 50	1.65 (−5.50 to 7.40)	5.6	C
PLT-I (×10^9^/L), 100	−1.88 (−4.70 to 0.37)	5.6	A
PLT-I (×10^9^/L), 600	−4.82 (−6.12 to −2.96)	5.6	C
PLT-I (×10^9^/L), 1,000	−5.05 (−6.55 to −2.89)	5.6	C

^*^Desirable allowable bias specifications were derived from the European Federation of Clinical Chemistry and Laboratory Medicine (EFLM) biological variation database (12). ^†^Outcome A indicates optimal agreement; Outcomes B–C indicate acceptable agreement; Outcomes D–E indicate unacceptable agreement according to CLSI EP09c criteria (7). HGB, hemoglobin; MCV, mean corpuscular volume; WBC, white blood cell count; PLT-I, impedance platelet count. 95% confidence intervals were derived from 1,000 bootstrap resamples. For some parameters with extremely narrow confidence intervals (e.g., HGB), lower and upper confidence limits may appear identical after rounding.

### Bland–Altman analysis

**Figure 2:**
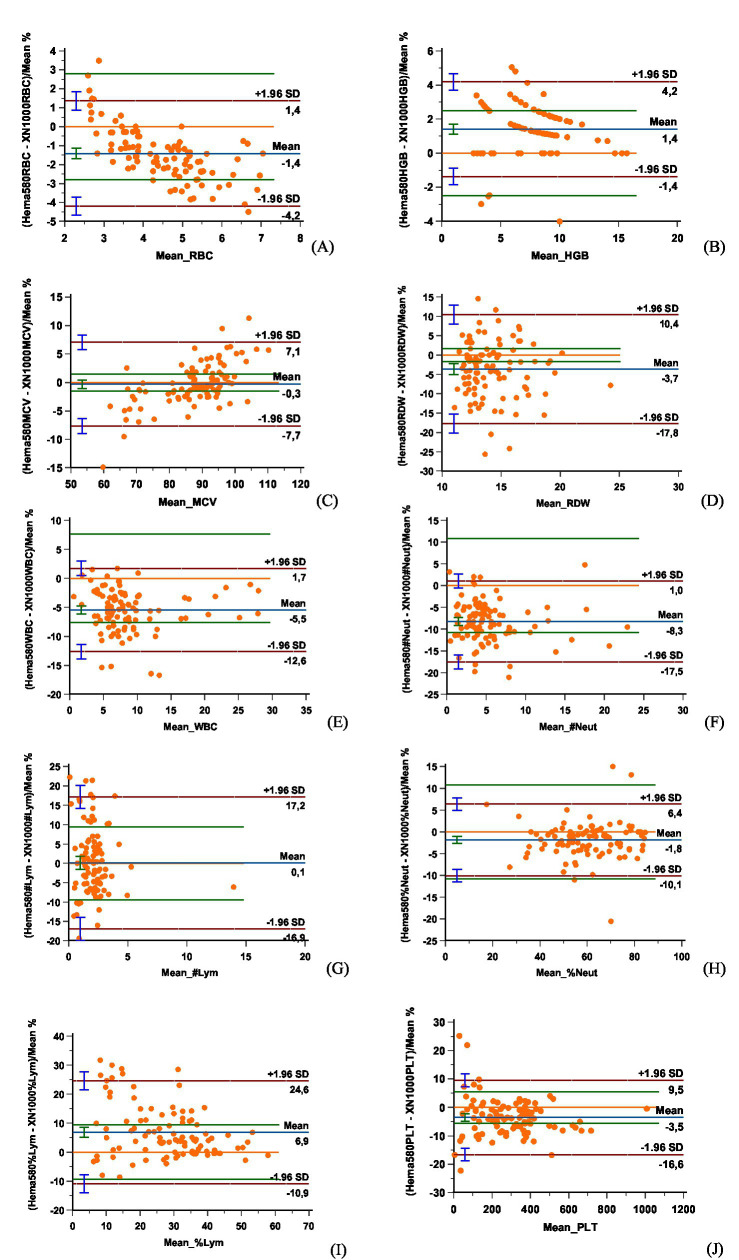
Bland–Altman plots showing agreement between the Atellica HEMA 580 and Sysmex XN-1000 analyzers for routine complete blood count (CBC) parameters. Panels show comparisons for **(A)** RBC, **(B)** HGB, **(C)** MCV, **(D)** RDW-CV, **(E)** WBC, **(F)** neutrophil count, **(G)** lymphocyte count, **(H)** neutrophils (%), **(I)** lymphocytes (%), and **(J)** PLT-I. The central solid line represents the mean bias, and the upper and lower dashed lines represent the 95% limits of agreement.

As was shown in Figure 2, Bland–Altman analysis demonstrated mean bias values and 95% limits of agreement (LoA) for all evaluated parameters. For RBC, the bias was −1.475% (95% CI: −1.799 to −1.267) with LoA ranging from −4.191 to 1.36%. HGB showed a bias of 1.54% (1.27 to 1.688) with LoA from −1.372 to 4.189%. MCV had a bias of −0.6% (−1.228 to −0.072) with wider LoA from −7.663 to 7.08%, while RDW-CV demonstrated a bias of −3.683% (−5.107 to −2.259) and the broadest LoA from −17.76 to 10.38%. WBC showed a bias of −5.195% (−6.158 to −4.241) with LoA from −12.62 to 1.698%. Neutrophil count had a bias of −8.267% (−9.205 to −7.328) with LoA from −17.54 to 1.003%, whereas lymphocyte count showed a bias of −1.115% (−3.274 to 0.729) with LoA from −16.94 to 17.19%. For differential percentages, neutrophils had a bias of −1.235% (−2.642 to −0.75) with LoA from −10.065 to 6.39%, and lymphocytes showed a bias of 4.32% (3.437 to 6.34) with LoA from −10.91 to 24.63%. PLT-I demonstrated a bias of −3.795% (−5.161 to −2.432) with LoA from −16.55 to 9.51%. Across parameters, data points were distributed around the zero-bias line with varying widths of agreement intervals.

### Bias assessment based on Bland–Altman estimates

Table 3 summarizes the Bland–Altman bias estimates, limits of agreement (LoA), and analytical performance classification for all evaluated parameters. According to the Shapiro–Wilk test, RDW-CV (*p* = 0.3201) and neutrophil count (*p* = 0.4221) demonstrated normal distributions, whereas the remaining parameters showed non-normal distributions (*p* < 0.05). Overall, most CBC parameters demonstrated acceptable analytical agreement according to CLSI EP09c criteria, with Outcomes A or B observed for all parameters except RDW-CV. The smallest mean bias was observed for lymphocyte count (−1.12%), which was classified as Outcome A, whereas RDW-CV showed the largest relative deviation (−3.68%) and was classified as Outcome E, indicating unacceptable agreement. MCV demonstrated a mean bias of −0.60% (95% CI: −1.23 to −0.07) with 95% LoA ranging from −7.66 to 7.08% and was classified as Outcome B despite the presence of systematic bias identified in Passing–Bablok regression analysis. This finding suggests that although average agreement for MCV remained within clinically acceptable limits in Bland–Altman analysis, proportional and constant bias across the measurement range may still affect clinical interchangeability at specific decision thresholds. WBC, neutrophil count, neutrophil percentage, and PLT-I showed moderate negative bias but remained within predefined allowable analytical limits. In contrast, RDW-CV demonstrated relatively wide limits of agreement and the poorest analytical performance among all evaluated parameters, supporting the observation of greater inter-analyzer variability for erythrocyte distribution indices. The highest acceptable bias thresholds ranged from 1.5 to 10.8% depending on the parameter.

**Table 3:** Bland–Altman bias estimates and limits of agreement for routine CBC parameters.

Parameter	Mean bias, % (95% CI)	95% limits of agreement	Desirable allowable bias*	CLSI EP09c outcome†	Shapiro–Wilk *p*-value (distribution)
RBC (×10^12^/L)	−1.48 (−1.80 to −1.27)	−4.19 to 1.36	2.8	B	0.0325 (non-normal)
HGB (g/dL)	1.54 (1.27 to 1.69)	−1.37 to 4.19	2.5	B	0.0001 (non-normal)
MCV (fL)	−0.60 (−1.23 to −0.07)	−7.66 to 7.08	1.5	B	0.0075 (non-normal)
RDW-CV (%)	−3.68 (−5.11 to −2.26)	−17.76 to 10.38	1.7	E	0.3201 (normal)
WBC (×10^9^/L)	−5.20 (−6.16 to −4.24)	−12.62 to 1.70	7.6	B	0.0070 (non-normal)
Neutrophil count (×10^9^/L)	−8.27 (−9.21 to −7.33)	−17.54 to 1.00	10.8	B	0.4221 (normal)
Lymphocyte count (×10^9^/L)	−1.12 (−3.27 to 0.73)	−16.94 to 17.19	9.4	A	0.0082 (non-normal)
Neutrophils (%)	−1.24 (−2.64 to −0.75)	−10.07 to 6.39	10.8	B	0.0001 (non-normal)
Lymphocytes (%)	4.32 (3.44 to 6.34)	−10.91 to 24.63	9.4	B	0.0001 (non-normal)
PLT-I (×10^9^/L)	−3.80 (−5.16 to −2.43)	−16.55 to 9.51	5.6	B	0.0001 (non-normal)

*Desirable allowable bias specifications were derived from the European Federation of Clinical Chemistry and Laboratory Medicine (EFLM) biological variation database (12). ^†^Outcome A indicates optimal agreement; Outcomes B–C indicate acceptable agreement; Outcomes D–E indicate unacceptable agreement according to CLSI EP09c criteria (7). CBC, complete blood count; CI, confidence interval; LoA, limits of agreement; HGB, hemoglobin; MCV, mean corpuscular volume; RDW-CV, red cell distribution width coefficient of variation; WBC, white blood cell count; PLT-I, impedance platelet count.

## Discussion

This study provides a comprehensive evaluation of analytical agreement between the Atellica HEMA 580 and the Sysmex XN-1000 under routine clinical laboratory conditions using the CLSI EP09c framework. Across the majority of parameters, the two analyzers demonstrated a high degree of concordance, as evidenced by strong Spearman’s rank correlation coefficients (*ρ* > 0.98 for most evaluated parameters), regression slopes close to unity, and minimal intercept deviation. Because correlation analysis reflects association rather than analytical agreement, correlation coefficients were interpreted only as supplementary indicators of comparability. The primary assessment of agreement was based on Passing–Bablok regression, bias estimation at predefined medical-decision levels, and Bland–Altman analysis in accordance with CLSI EP09c recommendations. These findings suggest that systematic differences between the two measurement systems are limited across the tested analytical ranges. Importantly, the consistency observed in this study aligns with previous evaluations of contemporary hematology platforms, which have similarly reported strong agreement for core CBC parameters, including RBC, HGB, WBC, and platelet indices (2, 4, 13). Comparable performance has also been described in studies involving analyzers based on similar impedance and fluorescence flow cytometric technologies, such as the Beckman Coulter DxH 800 (14). Collectively, these observations reinforce the concept that modern hematology analyzers built on comparable measurement principles can provide functionally interchangeable results for routine clinical use, particularly when analytical verification is performed prior to implementation.

Notwithstanding this overall agreement, the present study identified systematic differences for selected erythrocyte indices, most notably MCV. The presence of both constant and proportional bias indicates that inter-instrument differences are not uniform across the measurement range. Specifically, the observed regression pattern suggests a tendency for lower MCV values reported by the Atellica HEMA 580 at the lower end of the spectrum and relatively higher values at the upper end. Importantly, this finding was further supported by bias estimation at predefined medical-decision levels, where MCV exceeded the desirable allowable bias at both 80 fL and 100 fL and was classified as Outcome D according to CLSI EP09c criteria. This pattern has important implications for clinical interpretation, as MCV is a key parameter in the morphological classification of anemia. Even small systematic deviations near clinically important decision thresholds (particularly 80 fL and 100 fL) may result in reclassification of patients between microcytic, normocytic, and macrocytic categories. Such reclassification could potentially influence subsequent diagnostic evaluation and clinical interpretation, particularly when MCV is used as an initial discriminator in anemia workup.

The present study was designed as an analytical method-comparison study rather than a clinical classification study. Because specimens were not specifically selected according to MCV classification categories and the distribution of samples around the 80 fL and 100 fL thresholds was not predefined, reliable estimation of reclassification frequency was beyond the scope of the current analysis. Future studies specifically designed to evaluate diagnostic classification agreement would be required to quantify potential misclassification rates.

Accordingly, although the overall analytical agreement between the two systems was acceptable, MCV values should not be considered fully interchangeable across platforms, particularly in longitudinal monitoring or multicenter studies in which serial measurements may be performed on different analyzers. From a laboratory implementation perspective, institutions replacing or introducing hematology analyzers should consider performing local verification studies for MCV before routine implementation. In addition, direct comparison of serial MCV measurements generated from different analyzer platforms should be interpreted with caution, particularly in longitudinal patient monitoring and multicenter studies. From a technical perspective, these differences likely reflect variations in measurement physics and signal processing. Although both analyzers employ impedance-based techniques for cell volume determination, differences in aperture geometry, sheath flow stabilization, pulse processing, and histogram reconstruction algorithms can influence the estimation of mean cell volume, particularly at the tails of the distribution (9, 10). In addition, analyzer-specific calibration strategies and proprietary data-processing algorithms may further contribute to systematic divergence in erythrocyte volume measurements. Such methodological heterogeneity is inherent to analyzer design and highlights the importance of method-specific validation when interpreting red cell indices. Such methodological heterogeneity is inherent to analyzer design and highlights the importance of method-specific validation when interpreting red cell indices. Similar observations have been reported in previous hematology analyzer comparison studies, where MCV demonstrated greater susceptibility to inter-instrument bias than primary CBC parameters despite otherwise acceptable analytical agreement (4, 14). Taken together, these findings emphasize that high statistical correlation alone does not necessarily imply clinical interchangeability for erythrocyte size-related parameters.

RDW-CV demonstrated the greatest variability among all evaluated parameters, with lower correlation and broader dispersion compared to other indices. This finding is consistent with previous studies and manufacturer evaluation reports, which have shown that RDW-related indices generally exhibit lower inter-analyzer comparability and greater analytical variability than directly measured CBC parameters (3, 4, 10). Unlike primary measured variables, RDW-CV is a derived metric that reflects the coefficient of variation of the erythrocyte volume distribution. As such, it is highly dependent on the underlying shape and width of the red cell histogram, which can vary according to differences in data acquisition, binning strategy, smoothing techniques, and algorithmic correction applied by different manufacturers. Consequently, even when absolute cell volumes are comparable, RDW-CV may diverge due to differences in how the distribution is mathematically represented. Clinically, RDW has gained increasing attention as a prognostic biomarker in a range of conditions, including iron deficiency anemia, chronic inflammatory states, cardiovascular disease, and critical illness. Therefore, the observed inter-instrument variability may have implications not only for cross-platform comparability but also for longitudinal patient monitoring, particularly in settings where patients are followed using different analytical systems.

Because RDW has been incorporated into prognostic models and risk-stratification frameworks in several clinical settings, including cardiovascular disease, chronic inflammatory disorders, and critical illness, inter-analyzer variability may influence interpretation of prognostic thresholds and risk categorization when results generated from different analytical platforms are compared. Consequently, caution is warranted when applying published RDW-based prognostic cutoffs across different hematology analyzers. Furthermore, the relatively poor analytical agreement observed for RDW-CV in the present study supports previous observations from hematology analyzer comparison studies that RDW measurements remain insufficiently standardized across manufacturers and analytical platforms. Laboratories implementing a new hematology analyzer should therefore verify RDW analytical performance locally and consider validation of laboratory-specific reference intervals and clinical decision limits before routine clinical use, particularly when RDW is incorporated into diagnostic or prognostic algorithms.

In addition to analytical factors, pre-analytical variables must also be considered when interpreting inter-instrument differences. It is well recognized that EDTA-anticoagulated blood samples undergo time-dependent morphological changes, including red cell swelling and membrane alterations, which can influence measured parameters such as MCV and RDW (15, 16). Although all samples in this study were analyzed within 4 hours of collection—generally considered an acceptable stability window—minor variations in storage duration, temperature fluctuations, or sample handling could still contribute to observed variability. Furthermore, biological variability inherent to patient samples, including differences in hematocrit, reticulocyte fraction, or red cell deformability, may interact with analyzer-specific detection mechanisms. These factors underscore the importance of strict pre-analytical standardization and highlight the inherent complexity of comparing analytical systems using real-world patient specimens.

Bias estimation at predefined medical-decision levels provided additional insight into the clinical relevance of inter-instrument differences. Most parameters demonstrated acceptable analytical performance within the EFLM-defined allowable bias limits (Outcomes A–C), supporting their suitability for routine clinical use. In particular, HGB, RBC, and WBC maintained stable bias profiles across clinically relevant concentration ranges, indicating consistent agreement at decision thresholds. Notably, WBC at 0.5 × 10^9^/L and PLT-I at 100 × 10^9^/L achieved Outcome A, reflecting excellent concordance at critical clinical levels such as leukopenia and thrombocytopenia. The observed performance at low WBC concentrations may be partially explained by the extended counting strategies employed by the Atellica HEMA 580 in low-count modes, which enhance statistical precision in leukopenic samples (2, 10). In contrast, MCV exceeded allowable bias limits at both 80 fL and 100 fL, highlighting the presence of proportional bias at clinically meaningful thresholds. Bland–Altman analysis further demonstrated that, while mean bias across the measurement range remained relatively small for most parameters, the width of the limits of agreement varied substantially. This apparent discrepancy between global agreement metrics and threshold-based classification reflects the complementary nature of these analytical approaches within the EP09c framework, emphasizing that statistical agreement does not always translate directly into clinical interchangeability at specific decision points.

Overall, the findings of this study indicate that the Atellica HEMA 580 provides analytical performance broadly comparable to that of the Sysmex XN-1000 for RBC, HGB, WBC, PLT-I, and the evaluated neutrophil and lymphocyte differential parameters, with clinically relevant differences primarily observed for MCV and RDW-CV. The magnitude of bias for most parameters falls within clinically acceptable limits and is unlikely to affect routine interpretation or patient categorization in the majority of clinical scenarios. However, the observed variability in MCV and RDW-CV suggests that caution is warranted when comparing results across different analyzer platforms, particularly in longitudinal follow-up or multicenter studies. In such contexts, consistency of analytical systems is critical to ensure reliable trend interpretation, and laboratories should preferentially use the same analyzer platform for serial measurements whenever possible.

This study has several limitations. First, although the sample size was consistent with CLSI EP09c recommendations for method-comparison studies and covered a broad analytical measurement range, the study was conducted at a single center and included a relatively modest number of specimens. Consequently, the distribution of some analytes may not fully reflect the diversity of patient populations encountered in other laboratory settings. In addition, laboratory-specific operational factors may differ across institutions and could influence analytical agreement estimates. These factors include differences in patient case-mix and the prevalence of hematological abnormalities, as well as variations in analyzer maintenance schedules, preventive servicing procedures, reagent lot characteristics, calibration practices, and local quality assurance programs. Because all measurements in the present study were performed under a single laboratory’s operating conditions, the observed agreement may not fully capture variability encountered in other healthcare settings. Second, only selected leukocyte differential parameters (neutrophil and lymphocyte counts and percentages) were included in the predefined analytical comparison, and a complete five-part leukocyte differential evaluation was not performed. Consequently, agreement between the two analyzers for monocyte, eosinophil, and basophil measurements could not be established. Because these cell populations typically occur at lower concentrations and may demonstrate greater analytical variability, future studies incorporating larger sample sizes and enriched representation of abnormal differential counts are warranted to determine their inter-analyzer comparability. Third, although pre-analytical conditions were standardized, residual biological and handling-related variability inherent to patient samples may have influenced certain parameters, particularly those derived from distribution-based measurements such as RDW-CV.

## Conclusion

The Atellica HEMA 580 demonstrated acceptable analytical agreement with the Sysmex XN-1000 for RBC, HGB, WBC, PLT-I, and the evaluated neutrophil and lymphocyte differential parameters under CLSI EP09c evaluation, supporting its use in clinical hematology practice. Agreement was maintained across clinically relevant ranges for these parameters, whereas MCV and RDW-CV demonstrated analyzer-dependent differences that may affect cross-platform comparability and should therefore be interpreted with caution. These findings indicate that the Atellica HEMA 580 can be reliably implemented for routine testing, although careful interpretation is required when comparing red cell indices between systems, particularly in longitudinal follow-up or multicenter settings.

## Data Availability

The raw data supporting the conclusions of this article will be made available by the authors, without undue reservation.
